# Effect of sintering parameters on the mechanical properties of monolithic zirconia

**DOI:** 10.15171/joddd.2019.038

**Published:** 2019

**Authors:** Caner Öztürk, Gülşen Can

**Affiliations:** ^1^Department of Prosthodontics, Faculty of Dentistry, Hatay Mustafa Kemal University, Hatay, Turkey; ^2^Department of Prosthodontics, Faculty of Dentistry, İstanbul Aydın University, Istanbul, Turkey

**Keywords:** Flexural strength, monolithic zirconia, sintering parameters, surface roughness

## Abstract

***Background.*** Zirconia restorations with high mechanical properties are the current treatment options for fixed restorations with advantages of high biocompatibility and low pulp irritation. Although the effect of sintering time and temperature on the optical and mechanical properties of zirconia core material were investigated, the effect of these parameters on the translucent monolithic zirconia is still uncertain. This study aimed to evaluate the effect of the changes in sintering temperature and holding time on the mechanical and structural properties of monolithic zirconia.

***Methods.*** Totally, 340 self-colored (A2) zirconia specimens from two different monolithic zirconia groups (n=170) were prepared, measuring 15.5×12.5×1.2 mm. Then, 17 subgroups (n=10), including the control groups, were sintered according to sintering parameters. XRD analysis was used to determine phase transformations. The surface roughness of the specimens was evaluated using profilometry, and the flexural strength of the specimens was evaluated by the three-point bending test. The data were analyzed using three-way ANOVA and post hoc multiple comparison test with Bonferroni correction (a=0.05) at a significance level of 0.05. Independent-samples t-test was used to compare the subgroups between the control groups (P˂0.05).

***Results.*** No tetragonal-to-monoclinic phase transformation was observed in the groups. Changes in the sintering parameters did not significantly affect the surface roughness and flexural strength of monolithic zirconia. Surface roughness values for all the subgroups were above the clinically critical limit.

***Conclusion.*** According to the results of this study, changes in the sintering parameters did not affect the surface phase transformation, surface roughness, and flexural strength of monolithic zirconia.

## Introduction

Concurrent with the development of CAD/CAM technology, zirconia has been widely used in the dental field due to its excellent mechanical properties, high biocompatibility, and low allergic potential.^1^ Zirconia, without a glass component, is a polycrystalline polymorphic material and occurs in three forms of monoclinic, tetragonal, and cubic. Pure zirconia occurs in monoclinic phase at room temperature, becomes tetragonal between 1170ºC and 2370ºC, and the cubic phase is stable up to the melting point of 2680ºC.^2^ The yttrium-stabilized tetragonal zirconia polycrystalline (Y-TZP), the most commonly used zirconia in dentistry and stable with yttrium in nanometers at room temperature, could be obtained by alloying zirconia with 2‒3% of yttrium oxide.^3,4^

In previous studies, the flexural strength of zirconia ceramics varied between 608 and 1540 MPa, and these differences occur depending on sintering parameters, surface treatments, and microstructure.^5-7^ Stawarzcyk et al^7^ reported that an increase in the sintering temperature resulted in a decrease in the flexural strength of zirconia. In contrast, some studies reported that sintering parameters did not affect the flexural strength of zirconia ceramics.^8,9^

According to the literature, the critical smoothness for plaque accumulation is 0.2 μm.^10,11^ Heintze et al^12^ reported that rough surfaces reduce the fracture resistance of the material, and changes in surface roughness affect the wear properties of zirconia. In clinical applications, the grinding procedures made by the dentist also directly affect the wear properties of zirconia. Additionally, it has been reported that changes in surface roughness also stimulate phase transformation.^13^ Preis et al^14^ reported that the polishing process reduces surface roughness and phase transformations on the zirconia surface. Currently available zirconia materials are generally sintered between 1350ºC and 1600ºC, and yttrium is incorporated into the structure at high sintering temperatures.^15^ However, sintering temperatures higher than 1600ºC cause excessive grain growth and increase porosity. Besides, it was observed that the mechanical and optical properties of the zirconia were not sufficient at sintering temperatures of <1400ºC.^3,7^ The degradation of zirconia at low temperatures and the strength of the material depend on many factors, including the amount of the stabilizing oxide, the distribution of the stabilizer, the phase composition, the particle size and distribution, etching and surface treatments, and the presence of the secondary phases. Although these factors are independent, they are mainly affected by sintering parameters, which have been considered as predominant factors to obtain stable zirconia.^16^

Zirconia is used as an infrastructure due to its high opacity, and the main disadvantage of zirconia restorations is chipping in veneering ceramics. To overcome the chipping problem, the use of monolithic zirconia restorations, which can be used without veneering ceramics, has gained popularity. In addition, with monolithic zirconia blocks, it is possible to obtain more translucent and aesthetic restorations without veneering ceramics.^16^ Additionally, as a result of high flexibility and fracture strength, monolithic zirconia can be used even in cases where the interocclusal distance is insufficient in the posterior region.^3,7^

Changes in sintering parameters affect the properties and microstructure of zirconia.^17^ However, the effect of sintering parameters on monolithic zirconia, directly related to oral conditions, is still uncertain.^8,7,18^ Therefore, this study investigated the effect of sintering parameters on the microstructure, surface roughness, and flexural strength of monolithic zirconia materials. The null hypothesis of the study was that sintering parameters do not affect the microstructure, surface roughness, and flexural strength of the translucent monolithic zirconia.

## Methods

In this in vitro study, two different commercially available pre-sintered, self-colored (A2) translucent monolithic zirconia materials (Group TZI; Incoris TZI C, Sirona Dental Systems GmbH, Bensheim, Germany) (Group Up; Upcera, Shenzhen Upcera Co., Ltd, Shenzhen, China) were used. Totally, 340 samples (n=170) with a dimension of 19×15.5×1.6±0.05 mm were prepared from pre-sintered blocks using a precision cutting machine (Microcut 201 Mekto Instruments Inc., Istanbul, Turkey). All the samples in each group were randomly divided into 16 subgroups according to sintering temperature (1400ºC, 1450ºC, 1500ºC, and 1600ºC) and holding time (30, 60, 120, and 240 min) for each group (n=10). Additionally, one subgroup, sintered according to the manufacturer’s instructions, was defined as the control group (n=10) (Table 1). The heating and cooling rates were set to 10ºC/min. The sintering furnace (InFire HTC Speed, Sirona Dental Systems GmbH Bensheim, Germany) was calibrated before each sintering process according to the manufacturer’s instructions. The temperature changes were checked by the internal thermometer of the sintering furnace. After the sintering process, the dimension of all samples was measured using a digital micrometer, and the final dimension of the samples was 15.5×12.5×1.2±0.03 mm. Then, all the samples were thermocycled in a thermal cycling machine (Thermocycler THE-1100 SD Mechatronic GMBH, Feldkirchen, Germany), consisting of 10000 cycles at 5ºC and 55ºC with 20-second dwell time. After the thermocycling procedure, all the groups were ultrasonically cleaned with isopropanol solution and kept at room temperature in dry air.

**Table 1:** Sample size of the subgroups in terms of the holding time and sintering temperature

**Group TZI (n)**	**30 min.**	**60 min.**	**120 min.**	**240 min.**	**Total**
**1400ºC**	10	10	10	10	40
**1450ºC**	10	10	10	10	40
**1500ºC**	10	10	10	10	40
**1600ºC**	10	10	10	10	40
**Control Group**					10
**Total**					170
**Group UP (n)**	**30 min.**	**60 min.**	**120 min.**	**240 min.**	**Total**
**1400ºC**	10	10	10	10	40
**1450ºC**	10	10	10	10	40
**1500ºC**	10	10	10	10	40
**1600ºC**	10	10	10	10	40
**Control Group**					10
**Total**					170

### Microstructural analysis (XRD Analysis) 

The effect of sintering temperature and holding time on the phase composition and t-m transformation were crystallographically examined using a diffractometer (Bruker D8 Advance, Bruker AXS GmbH, Karlsruhe, Germany) on three randomly selected samples from each group. All the samples were subjected to Cu K(alpha) radiation. The voltage and current were set to 40 kVp and 40 mA, respectively. Diffraction profiles were recorded within a range of -10 to 90º, a continuous θ/2θ scan with a step size of 0.05º, and a scan speed of 4.0 deg/min.

### Surface roughness 

The surface roughness (Ra) of the groups was evaluated by using a contact profilometer (Perthometer M2, Mahr GmbH, Göttingen, Germany). Ra values of each specimen were obtained by three measurements on the three different axes passing through the center of the specimens, and mean Ra values were calculated. Measurement parameters were set to nOc: × 5; Oc/L: 0.8; range: 20×5. The profilometer was calibrated before each measurement.

### Flexural strength

A three-point bending test was performed to determine the flexural strength of the groups using a universal testing machine (LRX, Lloyd Instruments Ltd., Hampshire, UK) at a crosshead speed of 1 mm/min. The support distance was set to 8.5 mm, and the loading rod was 2 mm in diameter. The flexural strength was calculated according to the following formula:

σ = 3Nl/2bd^2^

(σ = flexural strength, N = fracture load [kg/mm^2^], l = the distance between the supports [8.5 mm], b = width of the specimen [12.5 mm], d = thickness of the specimen [1.2 mm]).

### Statistical Analysis

The statistical analysis was performed using SPSS 20 (SPSS Inc., Chicago, IL, USA). Shapiro-Wilks test was used to determine whether the surface roughness and flexural strength data showed normal distribution. Homogeneity of the data was analyzed with Levene's test at the 0.05 significance level. The data were evaluated using three-way ANOVA and post hoc multiple comparison test with Bonferroni correction (a=0.05) at a significance level of 0.05. Independent-samples t-test was used to compare the subgroups between the control groups (P˂0.05).

## Results

XRD analysis revealed no monoclinic (m) phase on the surface of the analyzed specimens within groups. Only tetragonal (t) characteristic peaks were determined on the surface of the specimens.

**Figure 1:**
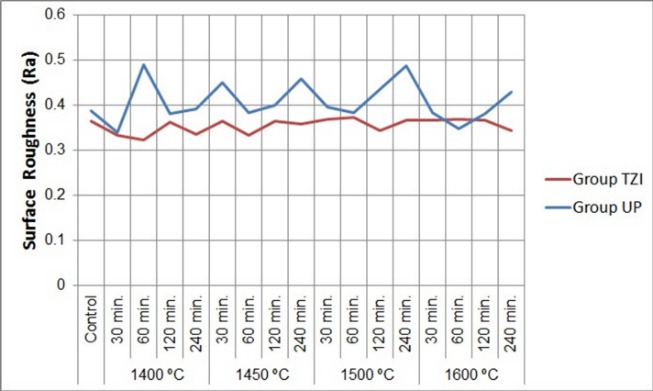
Surface roughness (Ra) changes as a result of changes in the sintering temperature and holding time

**Figure 2:**
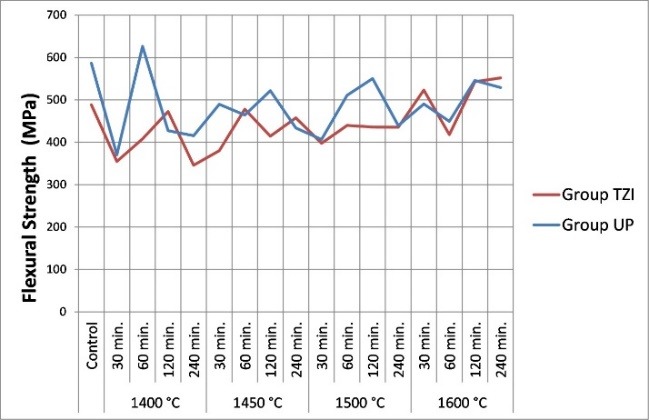
Flexural strength (MPa) changes due to sintering temperature and holding time.

According to the statistical analysis conducted, material, holding time, sintering temperature, and interaction between these factors were not significant for the surface roughness and flexural strength values (Tables 2 and 3). Surface roughness values (Ra) obtained from the groups are presented in Table 4 and Figure 1. No significant differences were found between the control group and subgroups in surface roughness values (P>0.05). The flexural strength values (MPa) obtained from the groups are presented in Table 5 and Figure 2. No significant differences were found between the control group and subgroups in flexural strength values (P>0.05).

**Table 2:** Univariate comparison of surface roughness (Ra) using three-way ANOVA

**Tests for inter-subject effects**					
**Surface roughness (Ra)**	**Type III Sum of Squares**	**df**	**Mean Square**	**F**	**Sig.**
**Intercept**	44.230	1	44.230	196.449	0.043
**Material**	0.223	1	0.223	39.771	0.339
**Sintering temperature**	0.033	3	0.011	2.409	0.613
**Holding Time**	0.023	3	0.008	0.891	0.635
Material * Sintering Temperature	0.022	3	0.007	0.553	0.659
Material * Holding Time	0.035	3	0.012	0.860	0.496
Sintering Temperature * Holding Time	0.096	9	0.011	0.785	0.638
Material * Sintering Temperature * Holding Time	0.122	9	0.014	1.851	0.059

**Table 3:** Univariate comparison of flexural strength (MPa) values using three-way ANOVA

	**Tests for inter-subject effects**				
**Flexural strength (MPa)**	**Type III sum of squares**	**df**	**Mean square**	**F**	**Sig.**
**Intercept**	54981711.760	1	54981711.760	310.425	0.001
**Material**	95426.642	1	95426.642	23.781	0.768
**Sintering Temperature**	216660.471	3	72220.157	2.852	0.252
**Holding time**	147434.274	3	49144.758	2.006	0.339
Material * sintering Temperature	45377.276	3	15125.759	0.595	0.634
Material * Sintering holding time	42920.883	3	14306.961	0.563	0.653
Temperature * holding time	320903.100	9	35655.900	1.402	0.312
Material * sintering temperature * holding time	228969.290	9	25441.032	1.421	0.180

**Table 4:** Mean and standard deviation values for surface roughness (Ra)

		**Surface Roughness (Ra)**	
		**Group TZI**	**Group Up**
**1400ºC**	**Control**	0.36±0.05	0.39±0.1
	**30 min.**	0.33±0.04	0.34±0.12
	**60 min.**	0.32±0.04	0.49±0.08
	**120 min.**	0.36±0.04	0.38±0.08
	**240 min.**	0.34±0.06	0.39±0.14
**1450ºC**	**30 min.**	0.36±0.06	0.45±0.13
	**60 min.**	0.33±0.07	0.38±0.05
	**120 min.**	0.36±0.05	0.40±0.11
	**240 min.**	0.36±0.05	0.46±0.11
**1500ºC**	**30 min.**	0.37±0.06	0.40±0.12
	**60 min.**	0.37±0.08	0.38±0.13
	**120 min.**	0.34±0.04	0.44±0.12
	**240 min**	0.37±0.04	0.49±0.09
**1600ºC**	**30 min.**	0.37±0.03	0.38±0.12
	**60 min**	0.37±0.07	0.35±0.11
	**120 min.**	0.37±0.06	0.38±0.13
	**240 min.**	0.34±0.07	0.43±0.02

**Table 5:** Mean and standard deviation values for Flexural Strength (MPa)

		**Flexural Strength (MPa)**	
		**Group TZI**	**Group Up**
	**Control**	488.50±87.53	586.54±236.32
**1400ºC**	**30 min.**	354.68±126.67	370.23±142.02
	**60 min.**	407.94±92.97	625.96±187.87
	**120 min.**	472.48±204.01	427.30±130.20
	**240 min.**	345.84±94.73	415.18±81.54
**1450ºC**	**30 min.**	379.96±123.24	489.61±186.61
	**60 min.**	478.05±177.25	464.12±102.60
	**120 min.**	414.21±99.34	521.62±179.75
	**240 min.**	457.53±103.81	433.50±56.88
**1500ºC**	**30 min.**	397.60±90.16	406.28±163.58
	**60 min.**	439.63±51.55	510.60±174.54
	**120 min.**	436.01±75.94	550.28±126.14
	**240 min**	435.34±118.11	439.15±79.73
**1600ºC**	**30 min.**	522.95±158.15	490.22±40.02
	**60 min**	417.98±51.12	449.28±134.27
	**120 min.**	542.80±192.52	545.71±102.84
	**240 min.**	551.77±119.94	529.04±171.32

## Discussion

According to the results of the study, the sintering temperature and holding time did not affect the microstructure, surface roughness, and flexural strength of the groups. Therefore, the null hypothesis of the study that sintering parameters do not affect the microstructure, surface roughness, and flexural strength of the translucent monolithic zirconia was accepted.

Contact profilometers are widely used in the dental field.^20,21^ These devices that provide surface-independent, high-resolution surface profile information, are scientifically accepted.^20^ Besides, contact profilometers have advantages of repeatability, applicability, and reliability. Therefore, the surface roughness of the specimens was measured using a contact profilometer. Changes in the surface roughness of materials affect the wear properties of zirconia restorations. This study showed that sintering conditions have no significant effect on the surface roughness of zirconia specimens. Consistent with the present study, Ebeid et al^9^ reported that the mean surface roughness value of zirconia decreased with an increase in sintering temperature and holding time, but these changes were not significant. Preis et al^14^ reported no significant difference in surface roughness between the zirconia specimens sintered with different sintering parameters, but the surface roughness of the specimens significantly decreased after glazing and polishing processes; therefore, phase transformation decreased on the surface of zirconia specimens. Surface roughness is associated with bacterial accumulation, and more bacterial involvement occurs on rough surfaces.^22^ Bollen et al^22^ reported that the roughness of tooth surface or any restoration should be <0.2 μm, and this value could be considered as a threshold value for bacterial involvement. The results of this study revealed that the surface roughness values for all the groups were >0.3 μm. These results show that polishing and glazing processes should be performed carefully for zirconia restorations after the sintering process or intraoral adjustment. Phase transformations on the zirconia surface negatively affect the mechanical properties of the material. Mechanical properties of zirconia and stability of the tetragonal phase depend on particle size and the metastable microstructure. In this study, sintering temperature and holding time did not affect the phase transformation on the surface of zirconia specimens. Consistent with the present study, Ebeid et al^9^ reported that changing the holding time and sintering temperature did not induce phase transformation on the monolithic zirconia surface. Hjerppe et al^8^ reported no phase transformation in the structure of the partially stabilized zirconia material with a decrease in sintering time. Contrary to the present study, Inokoshi et al^23^ investigated the effect of changes in sintering parameters on phase transformation in three different commercial zirconia materials and reported that the cubic phase increased in surface structure due to an increase in sintering temperature and holding time. The authors also reported that as a result of unstable zirconia microstructure, the monoclinic phase was determined at the highest sintering temperature, and changes in sintering parameters caused phase transformation. Differences between the present study and the study conducted by Inokoshi et al^23^ could be attributed to higher sintering temperatures used in the previous study.

Zirconia is the most durable ceramic material used in dentistry due to its high flexural strength.^2,4^ According to the results of this study, sintering temperature and holding time did not significantly affect the flexural strength of zirconia specimens. Consistent with the present study, Hjerppe et al^8^ reported that changes in sintering parameters and the thermal aging process did not affect the flexural strength of zirconia. Ebeid et al^9^ concluded that sintering parameters did not significantly affect the flexural strength of the translucent monolithic zirconia material. Contrary to the results of this study, Inokoshi et al^23^ reported that changing the sintering parameters affected the mechanical properties of the zirconia material. Trunec^24^ reported that the fracture strength of zirconia depends on the particle size that varies due to changes in sintering parameters. Stawarczyk et al^7^ concluded that the flexural strength of zirconia decreased with an increase in sintering temperature, and the highest strength could be obtained between 1400ºC and 1550ºC, and the lowest strength at low temperatures. These differences from other studies could be related to the structure of the material used, sintering parameters, and phase transformation of the material’s structure. In this study, the sintering parameters were determined at a certain sintering temperature and holding time intervals in accordance with the literature^7-9,18,19,23,25^ and manufacturer recommendations. Thus, further studies are needed to examine the effect of the sintering process at higher or lower temperatures and in shorter or longer holding times on the properties of zirconia. In addition, the effect of other sintering parameters such as heating rate and sintering atmosphere on the properties of the zirconia should be investigated.

## Conclusion

Within the limitations of our study, it was concluded that sintering parameters did not significantly affect the microstructure of translucent monolithic zirconia. Changes in the sintering parameters did not significantly affect the surface roughness of the translucent monolithic zirconia. For all the groups, the surface roughness was above the clinically acceptable values. Changes in sintering parameters did not significantly affect the flexural strength of the translucent monolithic zirconia.

## Authors’ contributions

CÖ and GC were responsible for the concept and the design of the study. CÖ performed the experimental design of study, data collection and statistical analysis. CÖ and GC were responsible for drafting the manuscript, critical revision of the article and final approval of the version to be published. All the authors participated in the literature review.

## Acknowledgments

None.

## Funding

This study was not supported financially by any individual or organization.

## Competing Interests

The authors declare no competing interests with regards to the authorship and/or publication of this article.

## Ethics approval

Not applicable.
